# *In vitro* evaluation of the sealing ability of three newly developed root canal sealers: A bacterial microleakage study

**DOI:** 10.4317/jced.52992

**Published:** 2016-12-01

**Authors:** Farnaz Jafari, Ehsan Sobhani, Hossein Samadi-Kafil, Ahmad Pirzadeh, Sanaz Jafari

**Affiliations:** 1 Assistant Professor, Endodontics Department, Dentistry Faculty, Tabriz University of Medical Sciences; 2 Dentist, Dentistry Faculty, Tabriz University of Medical Sciences; 3 Assistant Professor, Drug Applied Reserach Center, Tabriz University of Medical Sciences, Tabriz, Iran; 4 Assistant Professor, Oral health and community Dentistry Department, Dentistry Faculty, Tabriz University of Medical Sciences; 5 Assistant Professor, Orthodontics Department, Dentistry Faculty, Ilam University of Medical Sciences, Ilam, Iran

## Abstract

**Background:**

The purpose of this study was to compare the sealing ability of MTA Fillapex, Apatite Root Canal Sealer and AH26 sealers.

**Material and Methods:**

The present *in vitro* study was carried out on 142 extracted single-rooted human mature teeth. The teeth were randomly divided into three experimental groups (n=44) and two control groups (n=5). Three root canal sealers were MTA Fillapex, Apatite Root Canal Sealer and AH26. The teeth in the control groups were either filled with no sealer or made completely impermeable. The root canals were prepared and obturated with gutta-percha and one of the sealers. The teeth were sterilized with ethylene oxide gas prior to the bacterial leakage assessment using *Enterococcus faecalis*. Leakage was evaluated every 24 hours for 90 days. Data were analyzed with descriptive statistical methods and chi-squared test. If the data were significant, a proper post hoc test was used. Statistical significance was set at *P*<0.05.

**Results:**

The positive control specimens exhibited total bacterial penetration whilst the negative control specimens showed no evidence of bacterial penetration. At the end of the study, the analysis of microleakage with chi-squared test showed no significant differences between the experimental groups (*P*<0.05). The results of chi-squared test analyzing the pair-wise differences between the groups considering the numerical values for leakage day indicated the lowest leakage with AH26 and the highest with Apatite root sealer.

**Conclusions:**

According to the results of the present study, sealing ability of AH26 was significantly higher than that of MTA Fillapex and Apatite Root Canal Sealer.

** Key words:**Mineral Trioxide aggregate, root canal obturation, dental seal.

## Introduction

Bacterial microleakage is responsible for the majority of endodontic failures due to inadequate apical seal. MTA Fillapex sealer consists of natural resin, diluent resin, salicylate resin, mineral trioxide aggregate, silica nanoparticles and bismuth oxide based on manufactures’ claims. Sankin Apatite, a bioceramic sealer (1), contains a powder composed of alpha-tricalcium phosphate and hydroxyapatite, with polyacrylic acid as its liquid. This sealer is cytotoxic to some degrees (2).

Sankin Apatite is mixed with bismuth carbonate and iodoform so that it becomes visible on radiographs (3).

AH26 is an epoxy resin sealer. In a study by Oliveira (4), MTA Fillapex provided better bacterial seal compared to AH Plus. In a study by Sonmez *et al.* (5) MTA Fillapex exhibited more microleakage than AH Plus, with the use of dye microleakage method. In a study by Al-Haddad (1), Apatite sealer exhibited better adaptation with canal walls compared to AH Plus and MTA Fillapex.

Any new material introduced as an endodontic sealer should be studied for its sealability. Therefore, the purpose of the present study was to compare bacterial microleakage of three sealers, including MTA Fillapex, Apatite Root Canal Sealer and AH26 using *Enterococcus faecalis* bacterial strain, which is the most common cause of endodontic treatment failures.

## Material and Methods

The present *in vitro* study was carried out on 142 freshly extracted single-rooted human maxillary canines, extracted for perio-dontal reasons, with an average length of approximately 21 mm (~0.02). The design of the present investigation was approved in Research Vice-Chancellor, Tabriz University of Medical Sciences, Dentistry Faculty’s research committee (No. 56/5769). The root canals were shaped using 30/0.06 and 30/0.08 RaCe rotary files (FKG, La-Chaux De Fonds, Switzerland). The specimens were examined under an optical microscope at ×40 for the presence of cracks. Copious irrigation was carried out using 0.5% sodium hypochlorite and normal saline solutions. The smear layer was removed using 5% sodium hypochlorite and 17% EDTA. The teeth were randomly divided into three experimental groups (n=44) and 2 control groups (n=5). Then, the root canals were filled with gutta-percha (Diadent) and the relevant sealer in each group: MTA Fillapex (Angelus, Londrina, PR Brazil), Apatite Root Canal Sealer (Sankin-Kogyo, Tokyo, Japan) and AH26 (DeTrey, Dentsply, Konstanz, Germany) with lateral condensation technique.

The sample size was considered using a five-sample scale by a pilot study. Total sample size was calculated at 132 teeth which was estimated by comparing two averages of 0.05 and a power of 80%.

In the positive control group, gutta-percha without sealer was used as a filling material for the root canal. The teeth were stored for 48 hours at 37ºC and 100% humidity. Then, 2 layers of nail varnish were applied on all the root surfaces except for 2 mm of the root end. In the negative control group, all the surfaces were covered with 2 layers of nail varnish.

The teeth were placed inside 1.5-mm Eppendorf plastic tubes (Elkay, Shrewsbury, MA, USA). Penicillin vial caps were used to secure the connection between the Eppendorf tube and the teeth. The whole system was then sterilized by ethylene oxide gas for 12 hours and placed in a sterile glass flask containing 6 mL of BHI (BHI-Oxid LTD, Hanks, USA) with 2 mm of root end in BHI. The connection areas of leakage assessment flask were sealed with cyanoacrylate glue.

The rest of the samples were placed within the Eppendorf tubes and each was filled daily with *E. faecalis* to optical density of 550 nm. The whole system was incubated for 90 days at 37ºC and the turbid solutions were recorded as positive leakage. Medium turbidity in the lower chamber was a strong indicator of contamination with the microorganism. Turbid lower chamber content cultured in order to ensure absence of cross contamination.

-Statistical Analysis

Data obtained from the study was analyzed using descriptive statistics (frequency, percentage), chi-squared test and in case of significance, with an appropriate post hoc test. IBM SPSS Statistics 17.0 was used to analyze data. In this study, statistical significance was set at *P*≤0.05.

## Results

In this study, 142 maxillary canines were used (44 teeth in each experimental group and five teeth in each control group). None of the examined samples were excluded due to the failure of seal or cracks.

None of the negative control samples showed bacterial penetration and leakage was seen in all the positive control group samples. Culturing lower chamber did not show cross contamination with bacteria other than *E. faecalis.*

Repeated-measures ANOVA was used to estimate the leakage changes between the experimental groups for the longitudinal analysis of test results, which showed no differences between the sealers in 90 day (*P*=0.263).

**Figure 1:**
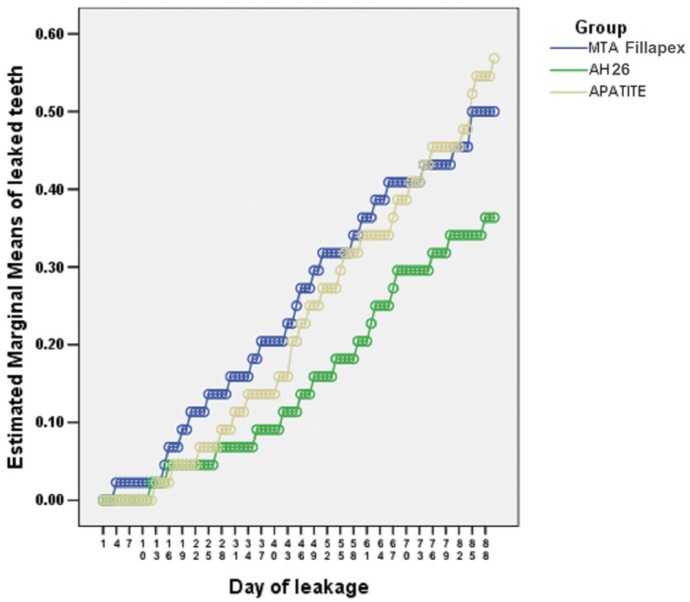
The results of ANOVA and post hoc tests for two-by-two comparisons of bacterial microleakage of experimental groups.

Figure 1 shows the mean leakage day of each experimental sample during the study period in the experimental groups.

Post hoc tests were used for two-by-two comparisons of all the materials.

The differences in the increasing microleakage of MTA Fillapex with Apatite and AH26 sealers were not significant (*P*=0.111 and *P*=0.691, respectively).

In addition, the difference between AH26 and Apatite was not significant (*P*=0.229).

-Chi-squared test to estimate the percentage and frequency of all the samples with leakage at the end of 90 days

Chi-squared test was used to analyze groups cumulatively and separately. Chi-squared test showed that at the end of 90 days, in the MTA Fillapex group 50% of the samples (22 out of 44 teeth) had leakage. In the AH26 group, 36.3% (16 out of 44) and in the Apatite Root Canal Sealer group 56.8% of the samples (25 out of 44) were positive in terms of leakage and there were no significant differences between the groups (*P*=0.14).

-Survival Analysis

In all the samples and all the time intervals, microleakage of AH26 was less than the other groups. The microleakage of MTA Fillapex was more than that of Apatite up to the 70th day. However, after 80 and 90 days, there was less microleakage with Apatite.

Kaplan-Meier method was used to analyze the mean survival of the samples. Plotting with the log-rank test was used to compare survival functions and to evaluate the survival rate in the three groups.

**Figure 2:**
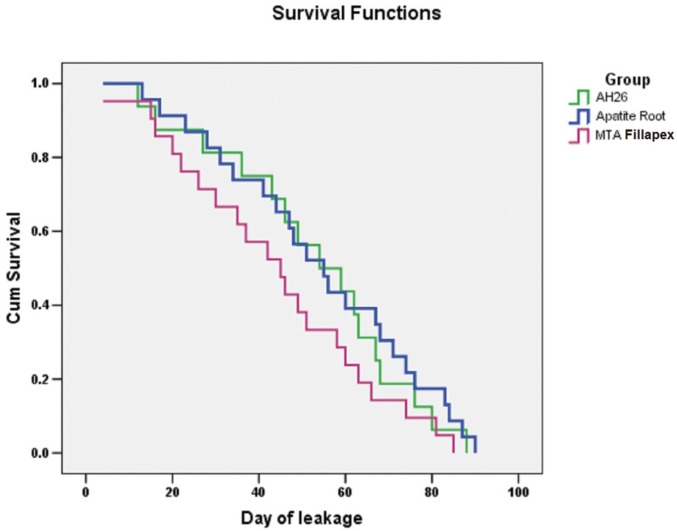
Diagram of analysis of survival probability of groups evaluated for 90 days.

The results of log-rank test did not show statistically significant differences between the three groups in terms of survival rate (*P*=0.254). The results of the survival analysis are shown in figure 2 in term of the survival of the groups in 90 days.

-Chi-squared test to calculate the percentage of leaked samples by assigning numerical values to leakage day

Chi-squared test was used to calculate the leakage in each group by assigning a numerical value to the leakage day because the leakage day is numerically valuable, i.e. a sample that showed leakage on the 10th day was not numerically the same as the sample that exhibited leakage on the 80th day.

Based on the results presented in table 1 and in accordance with chi-squared test, in the Apatite group 25.1%, in the AH26 group 15.4% and in the MTA group 22.7% of the samples exhibited microleakage at the end of 90 days, with significant differences between the groups (*P*=0.001 and 120.7); the highest microleakage was recorded in the apatite group and the lowest in the AH26 group ( Table 1).

**Table 1:**
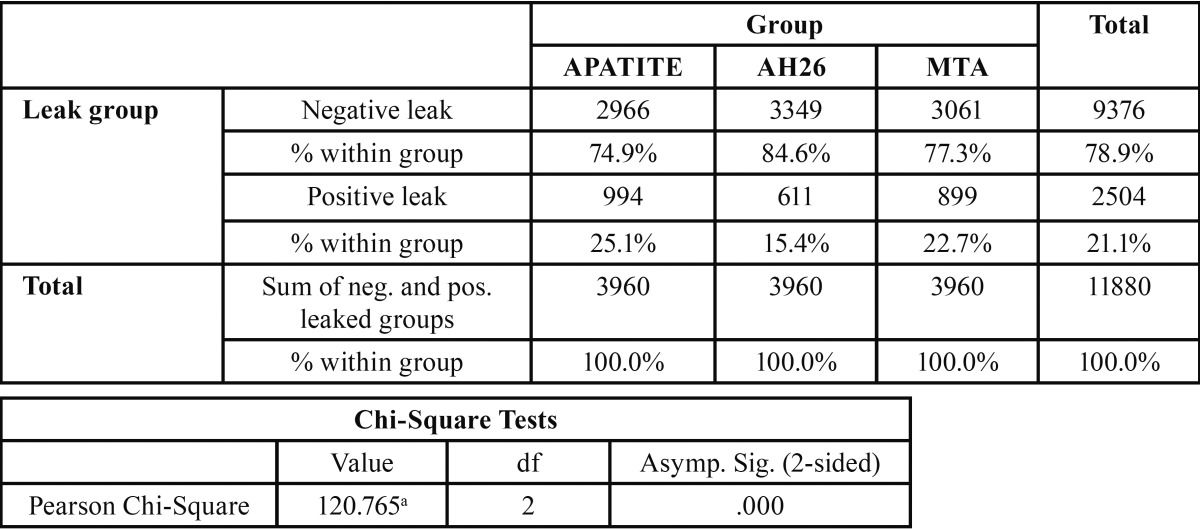
Chi-square test for experimental groups with assigning a numerical value for leakage day.

In each experimental group, within 90 days of the study, a numerical value equal to the day on which leakage took place was assigned to samples. Therefore, numerical value of 15 indicated microleakage on the 15th day.

For example, the total number of days without and with leakage for these teeth were 15 and 75 days, respectively. The numerical value of the study data calculated for each group was 90×44=3960.

-Two-by-two chi-squared analysis to calculate the leakage day of each group with numerical values for leakage day

There was a significant difference between AH26 and Apatite, with AH26 exhibiting superiority to Apatite because of lower leakage (*P*<0.001).

There was a significant difference between MTA Fillapex and Apatite. MTA Fillapex exhibited less leakage, indicating its superiority to Apatite (*P*=0.012).

There was a significant difference between MTA Fillapex and AH26. AH26 exhibited less leakage, indicating its superiority to MTA Fillapex (*P*< 0.001).

In general, according to the results of the present study, AH26 exhibited minimal leakage compared to the two other groups. Then MTA Fillapex and Apatite showed the least microleakage, respectively (*P*<0.05).

## Discussion

Achieving a proper seal is one of the most important goals of root canal treatment and different materials have different abilities to seal (6-13). *In vitro* studies for microleakage include dye leakage, radioisotopes and autoradiography to investigate electrochemical, infiltration and liquid emissions, scanning electron microscopy and microbial penetration (14).

The most commonly used technique is dye leakage because it is the easiest method to evaluate leakage, but bacterial leakage is preferable due to better clinical conditions and similarity to the oral cavity conditions and can be a good technique to check leakage with one or more bacterial strains that might be the cause of failure (15).

Since the major cause of root canal treatment failures is *E. faecalis* bacteria, this strain was used in the present study (16).

Selection criteria of canine tooth was the longest root and its minor root anatomical variations. The sample size was determined by a pilot study on a scale of 5 samples.

Van der Sluis *et al.* (17) showed that the rate of leakage was different in oval and round root canals. Therefore, this study was performed on single-rooted canines with straight roots because the roots of this teeth were long and had minor anatomical variations. The use of bacteria for microleakage assessment simulates clinical conditions (13).

The bacterial composition of previously treated canals with apical periodontitis includes a limited strain of bacteria compared to the initial infection and consists of an average of one to three species per canal. *E. faecalis* is a gram-positive anaerobic cocci, which is isolated from failed root canals at a prevalence rate of 30-90% (18,19).

There are doubts about the value of laboratory microleakage studies due to the clinical importance and the limitations of the results of these studies (20). Nevertheless, these studies are widely used to evaluate and compare the performance of sealers, i.e. the principal aim of the sealer in obturating the root canal, before their application in the clinic.

MTA Fillapex is a new MTA-based sealer. According to the manufacturers’, this MTA-based sealer creates integrated, excellent and perfect seal and provides high biological regeneration (21). However, more recent studies have shown conflicting results regarding this claim (21,22).

MTA setting leads to hydration of inorganic oxide compounds, resulting in the production of calcium hydroxide and calcium silicate hydrate phases (23), which in turn lead to expansion at its margins, improving the seal and reducing microleakage (24).

AH26 sealer was used in this study as a basis for comparison with other sealers because of variability of studies that have been carried out on this material. Tagger *et al.* (25) reported significant adhesion between AH26 and dentin. Lee *et al.* (26) showed that AH26 sealer exhibits better adhesion with dentin compared to Ketac Endo and Sealapex and even can form a covalent bond with collagen compounds.

Sonmez *et al.* (5) showed that MTA Fillapex has less sealing ability compared to AH Plus, and ProRoot MTA using the dye penetration method. In the present study, AH26 was used instead of AH Plus and it was concluded that AH26 had better seal compared to MTA Fillapex. The superiority of the present study over the stated research was a larger sample size and microleakage assessment.

To obtain statistically valid results, the study period was set at 90 days in the present research. In studies conducted by Roberts *et al.* (27) the period was 60 days. In the study by Yavari (13) it was 90 days period, although leakage in the last sample occurred in less than 60 days.

Another strong point of this study was the statistical analysis in this study which was different from previous studies because numerical values describe the situation closer to the real situation.

Nikhil *et al.* (28) compared the depth of penetration of MTA Fillapex and AH Plus sealers into the dentinal tubules with the use of a confocal laser microscope and reported that MTA Fillapex sealer penetrated deeper into the dentinal tubules. These results do not coincide with the results of the present study or it is better to conclude that although MTA Fillapex exhibits deeper penetration into dentinal tubules, it does not result in better apical seal. Such differences in the results might be attributed to differences in the methodologies of these studies.

Furthermore, the results of the present study coincide with those of another study that reported that bioceramic-based sealers had more gaps compared with AH Plus (1).

However, the results of this study coincide with those of a study by Reyhani *et al.* (29) on the bacterial microleakage of four different types of sealers after preparation of post spaces. Such a difference might be attributed to differences in sample sizes and also differences in methodologies of these studies.

Apatite Root Canal Sealer’s sealing ability was less than that of AH Plus (30). Despite differences in methodologies of these stu-dies the results coincide with those of the present study.

The results of the present investigation are consistent with those of another study which concluded that bioceramic-based sealers exhibited more gap-containing regions when compared with AH Plus (1).

The results of the present study are not consistent with those of a study by Reyhani *et al.* (29) on the bacterial microleakage of four different sealers after post space preparation. The reason for this contrast might be different sample sizes and also different methods and materials in these studies.

## Conclusions

AH26 sealer showed the lowest leakage compared to Apatite Root Canal Sealer and MTA Fillapex.
